# A high dimensional immune monitoring model of HIV-specific CD8 T cell responses accurately identifies subjects achieving spontaneous control of viral replication

**DOI:** 10.1186/1742-4690-9-S1-P19

**Published:** 2012-05-25

**Authors:** Zaza M Ndhlovu, Lori B Chibnik, Jacqueline Proudfoot, Seanna Vine, Ashley McMullen, Kevin Cesa, Donna Marie Alvino, Alicja Piechocka-Trocha, Philip L de Jager, Daniel E Kaufmann, Bruce D Walker

**Affiliations:** 1 Ragon Institute of Mgh, Mit and Harvard, Charlestown, USA

## Introduction

A major challenge in HIV vaccinology is the development of appropriate immune monitoring models to determine vaccine efficacy. Studies of HIV-specific CD8 T cells (CTL) suggest that these responses play an important role in infected individuals capable of spontaneous viral control (HIV elite controllers) and that they will likely play a role in immune interventions. However, no single CTL assay is uniquely associated with the controller phenotype.

## Method

We compared functionality of HIV-1-specific CTLs in individuals with spontaneous viral control and subjects with treated or untreated progressive infection. A model integrating multiple features of epitope-specific CTL responses that delineate HIV controllers from subjects with treated or untreated progressive infection was built.

## Results

Area Under the Receiver Operating Characteristic (ROC) Curve showed that proliferative capacity, absolute early cytokine production and kinetics of cytokine secretion were all associated with HIV control. However, only integrated modeling of these different dimensions of data allowed reaching the remarkable 90% accuracy, which was validated in separate cohorts.

## Conclusions

Our results suggest that while the search for a common determinant of protective immunity remains elusive, combining parameters generated by various well-established assays in models that can be iteratively refined may have important applications for predicting disease outcome and for immune monitoring of HIV-1 vaccine trials.

